# Major impact of moist wound healing on autologous tissue regeneration: A review of ulcer treatment

**DOI:** 10.1002/hsr2.1029

**Published:** 2022-12-31

**Authors:** Kenji Yamamoto, Senri Miwa, Tomoyuki Yamada, Shuji Setozaki, Mamoru Hamuro, Shunji Kurokawa, Sakae Enomoto

**Affiliations:** ^1^ Department of Cardiovascular Surgery Okamura Memorial Hospital Shizuoka Sunto‐gun Japan; ^2^ Department of Cardiovascular Surgery Shiga General Hospital Moriyama Shiga Japan; ^3^ Department of Cardiovascular Surgery Shizuoka General Hospital Shizuoka Shizuoka Japan

**Keywords:** dressings, finger amputation, microbiome, moist wound healing, tissue regeneration, ulcer

## 1 INTRODUCTION

Ulcers are frequently intractable and can be venous in origin or arise from injuries, such as finger amputations or burns. Venous ulcers are most common in the lower extremities, affecting approximately 1% of the United States population. Although clinical practice guidelines of the Society for Vascular Surgery recommend moist dressings for wound care, the rate of dressing usage, excluding gauze, in 2016 was <10% in Japan. Compared with conventional treatments such as disinfection and gauze, endovenous ablation of venous reflux; adequate varicose vein resection, including incompetent perforator veins^1^; and compression therapy with moist wound healing^2^ and appropriate skin care, yield more rapid epithelialization and prevent a recurrence. This study aimed to describe the widespread use of moist wound healing for venous ulcer treatment in Japan and its application to various wounds, particularly difficult‐to‐treat finger amputations and burns.

## 2 MATERIALS AND METHODS

The development of irreversible membrane (IRM) dressings (Plus Moist™; Zuiko Medical Osaka) and composite absorbent pads (Zuikopad™; Zuiko Medical Osaka) in Japan have improved the moist wound healing technique.^2^ Showering wounds twice daily, regardless of their possible infections, and covering them using these functional dressings could provide an optimal environment for tissue regeneration through excess exudate absorption and wet wound surface maintenance. Natsui reported ˃4000 cases where moist wound healing was used,^3^ reflecting its increasing popularity.^4^

## 3 RESULTS

### 3.1 Finger amputation

**Figure 1:**
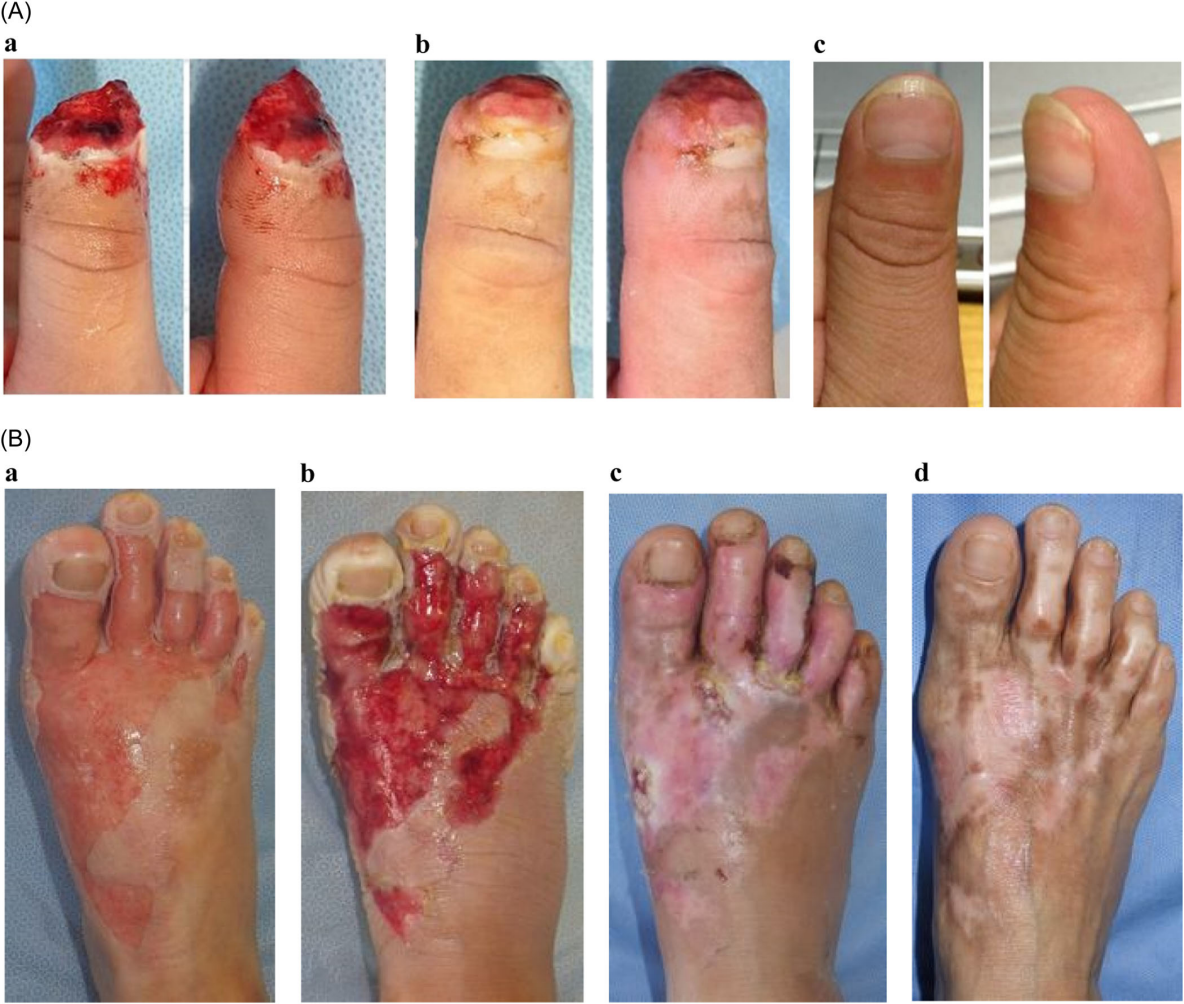
(A) Representative case of an injured patient treated with an irreversible membrane (IRM) dressing. These figures were acquired from http://wound-treatment.jp/next/case/459.htm after obtaining permission for their inclusion. A 25‐year‐old man underwent amputation of the tip of his left first finger. a: At the first visit. b: 3 weeks after the first visit. c: 5 months after the first visit, the regenerated fingertip shows no scar. (B) Representative case of a patient with a burn injury treated using an IRM dressing. These figures were acquired from http://www.wound-treatment.jp/next/case/tokyo/index.htm after obtaining permission for their inclusion. A 62‐year‐old woman with hot water burns (second‐degree burns) on the right foot. a: At the first visit. b: 6 days after the first visit. c: 1 month after the first visit. d: 4 and a half months after the first visit, the regenerated skin remains pigmented shows no scarring or joint contracture.

More than 110 finger amputation cases were presented from the ˃4000 reported cases, including minor cases where the apex part of the finger was amputated with a blade, contusions, and fractures and cases where vascular anastomosis or composite grafts were performed at several hospitals. Moist wound healing results in almost complete epithelialization without necessitating reamputation. Figure 1A shows a complete regeneration of finger tears that would have been indicated for amputation using conventional methods.^2^, ^5^ Some deformities and shortening of the fingers are unavoidable in major amputation cases; however, no complications were observed in moving the treated finger in most cases. Furthermore, no patient underwent skin grafting or vascular anastomosis in Natsui's study.^3^ Cases of conservative treatment of finger amputation using IRM dressings have also been reported.^6^

### 3.2 Burns

Natsui achieved epithelial regeneration without major joint contraction using moist wound healing in patients with burns, presenting many cases from actual clinical practice^3^ (Figure 1B). Moreover, many similar cases have been reported.^7^

## 4 DISCUSSION

Although regeneration took approximately 5 months in previous reports, no scarring was observed in completely regenerated fingertips. Finger amputation, managed surgically or conservatively (revision amputation), is more common in the United States.^6^

Moist wound healing has reduced the need for skin grafts and even treating third‐degree burns, despite requiring a longer treatment period. Traditionally, skin grafts are recommended for unhealed wounds for approximately 2 weeks after a burn injury. However, skin grafting is generally accompanied by the disfigurement of both the harvest and grafting sites. Moist wound healing could prevent joint contracture, even with hypertrophic scarring, by promoting the active movement of the wound, which preserves the joint's range of motion. In contrast, skin grafting is associated with unfavorable joint contractures and requires wound immobilization. Although epithelialization may take weeks to years, the risk of infection is lower for moist wound healing.^4^, ^8^ Generally, larger wounds take longer to heal.

Moist wound healing functions by maintaining a balanced microbiome in the sebum that contains approximately 1000 species of resident flora,^9^ mainly anaerobic bacteria, and several species of fungi. Therefore, maintaining the resident flora diversity and balance, rather than eliminating them through sterilization or disinfection, is important.^10^ If the flora is disrupted, *Staphylococcus aureus* and other aerobic bacteria may abnormally predominate, causing infection. Although the superiority of moist wound healing over other healing methods has been demonstrated in wound management, gauze remains widely used because of insufficient appropriate covering materials.

Dressing selection is important for moist wound healing. Moreover, the cost must be considered because of the requirement for twice‐daily changes during moist wound healing. Hydrocolloids are useful when there is little or no exudate. However, when exudate is present, the risk of infection increases if the wound is completely closed by the dressings. The IRM principle is simple: irreversible moderate absorption of leachate to preserve surface moisture, which contains growth factors. These regimens maintain microbiome balance, which is crucial for infection control.^11^

Negative pressure wound therapy (NPWT) has been used to compensate for excessive leachate and wound enlargement.^12^ However, NPWT should not be used with suspected infection. Although moist wound healing requires regular dressing replacement, the patient can still bathe, work, and maintain activities of daily living without hospitalization.

Along with dressing usage, infection control and pain management are important for wound management. Specifically, it is important to avoid removing excess sebum. Disinfectants (povidone‐iodine and silver sulfadiazine^13^), antibiotic ointments, soaps, and creams containing surfactants should be avoided. Additionally, trafermin is not used because it causes localized pain.^2^ Smoking and secondhand smoking are important risk factors for delayed wound healing and increased infection.^14^ Nonsteroidal anti‐inflammatory drugs and acetaminophen use should be limited since they can decrease body temperature and immunity.^15^ Compression therapy should be used for the lower legs since they are prone to gravity‐induced edema.

The surrounding tissues, including the epithelium, can regenerate by providing an appropriate moist environment with a balanced microbiome. Nevertheless, despite using moist wound healing, we have experienced several cases of epithelialization of venous ulcers after short‐term topical steroid use in patients with residual erosions.^2^ The rate of epithelial skin cell turnover is approximately 1 month.^16^ Although topical steroids have mitogenic effects on epithelial skin cells,^17^ they may disrupt the microbiome balance. Therefore, their long‐term use or use in ulcerated areas is not recommended.

Careful observation is necessary after using moist wound healing. Wounds that have undergone scar contraction due to disinfection and/or gauze use may temporarily enlarge during moist wound healing.^2^ Furthermore, early return to the outpatient clinic is recommended after moist wound healing if signs of infection appear, including exacerbated spontaneous pain, foul odor, or blackened granulation. Maintaining a moist wound environment frequently reduces pain. Therefore, oral or intravenous antibacterial agents should be administered if an infection is suspected. Furthermore, in the post‐messenger RNA vaccination era, the risk of infection in critically ill patients is increasing, necessitating more careful management while maintaining a moist environment.^18^

Appropriate moisturizing is necessary for maintaining normally regenerated skin. White petroleum jelly is generally recommended for patients receiving moist wound healing because of its safety and moisturizing qualities. However, it may cause patients to apply soap to eliminate greasiness, which could lead to increased irritation. This phenomenon has been named the “petroleum jelly paradox.” After Mirai Kankyo Technology succeeded in emulsifying petroleum jelly using a 3‐phase emulsification process where water and oil are mixed without surfactants,^2^ the authors began recommending a cosmetic moisturizer (Proud Blue Sensitive Moisture Cream™) developed using this technology. Meanwhile, ordinary cream formulations contain surfactants. The heparinoids used in recently popularized moisturizers present the risk of vaccine‐induced thrombotic thrombocytopenia.^19^

Wounds with contraindicated moist wound healing include those with complete skin necrosis (blackish brown) without a chance of recovery, such as in severe lower extremity ischemia cases.^20^

## 5 CONCLUSION

Moist wound healing maximizes the autologous regeneration of tissues by preserving the microbiota balance, which may be a valuable option for wound management.

## AUTHOR CONTRIBUTIONS

**Kenji Yamamoto**: conceptualization; writing – original draft. **Senri Miwa**: investigation; writing – review & editing. **Tomoyuki Yamada**: investigation; writing – review & editing. **Shuji Setozaki**: investigation; writing – review & editing. **Mamoru Hamuro**: investigation; writing – review & editing. **Shunji Kurokawa**: investigation; writing – review & editing. **Sakae Enomoto**: investigation; writing – review & editing. All authors have read and approved the final version of the manuscript.

## CONFLICTS OF INTEREST

The authors declare no conflicts of interest.

## ETHICS STATEMENT

This study was approved by the Institutional Review Board of Okamura Memorial Hospital (approval number: A019‐001), and the need for individual patient consent was waived. Informed consent forms were obtained, as applicable.

## TRANSPARENCY STATEMENT

The lead author Kenji Yamamoto affirms that this manuscript is an honest, accurate, and transparent account of the study being reported; that no important aspects of the study have been omitted; and that any discrepancies from the study as planned (and, if relevant, registered) have been explained.

## Data Availability

Data are available upon request to the corresponding author, Kenji Yamamoto, who had full access to all data in this study and takes full responsibility for the integrity of the data and the accuracy of the data analysis.

## References

[1] Yamamoto K , Miwa S , Yamada T , et al. Feasibility of incompetent perforator vein excision using stab avulsion. Phlebology: The Journal of Venous Disease. 2022;37:393‐399. 10.1177/02683555221081816 PMC9168896 35318865

[2] Yamamoto K , Miwa S , Yamada T , et al. A strategy to enable rapid healing and prevent recurrence of venous ulcer. Wounds. 2022;34:99‐105. 35452407

[3] Natsui M . Treatment course of all cases. Available from, (Accessed October 23, 2022). (written in Japanese). http://www.wound-treatment.jp/next/case/tokyo/index.htm

[4] Natsui M . Overview of wound healing in a moist environment. Available from, (Accessed October 23, 2022). www.wound-treatment.jp/english/index_e.htm

[5] Natsui M . Injury to the tip of the first finger. Available from, (Accessed October 23, 2022). (written in Japanese). http://wound-treatment.jp/next/case/459.htm

[6] Masaki S , Kawamoto T . Fingertip amputation injury of allen type III managed conservatively with moist wound dressings. American Journal of Case Reports. 2021;22:e928950. 33621217 10.12659/AJCR.928950 PMC7913779

[7] Masaki S , Maeda I , Kawamoto T . Conservative management of full‐thickness burn wounds using advanced moist dressings: a case report. Wounds. 2022;34:e42‐e46. 36075046

[8] Field CK , Kerstein MD . Overview of wound healing in a moist environment. Am J Surg. 1994;167(1A):S2‐S6. 10.1016/0002-9610(94)90002-7 8109679

[9] Gao Z , Perez‐Perez GI , Chen Y , Blaser MJ . Quantitation of major human cutaneous bacterial and fungal populations. J Clin Microbiol. 2010;48:3575‐3581. 20702672 10.1128/JCM.00597-10 PMC2953113

[10] Grice EA , Kong HH , Conlan S , et al. Topographical and temporal diversity of the human skin microbiome. Science. 2009;324:1190‐1192. 19478181 10.1126/science.1171700 PMC2805064

[11] Kunz TC , Kozjak‐Pavlovic V . Diverse facets of sphingolipid involvement in bacterial infections. Front Cell Dev Biol. 2019;7:203. 31608278 10.3389/fcell.2019.00203 PMC6761390

[12] Capobianco CM , Zgonis T . An overview of negative pressure wound therapy for the lower extremity. Clin Podiatr Med Surg. 2009;26:619‐631. 19778692 10.1016/j.cpm.2009.08.002

[13] Heyneman A , Hoeksema H , Vandekerckhove D , Pirayesh A , Monstrey S . The role of silver sulphadiazine in the conservative treatment of partial thickness burn wounds: a systematic review. Burns. 2016;42:1377‐1386. 27126813 10.1016/j.burns.2016.03.029

[14] Mekako AI , Chetter IC , Coughlin PA , Hatfield J , McCollum PT , Hull Antibiotic pRophylaxis in varicose Vein Surgery Trialists (HARVEST) . Randomized clinical trial of co‐amoxiclav versus no antibiotic prophylaxis in varicose vein surgery. Br J Surg. 2010;97:29‐36. 20013927 10.1002/bjs.6849

[15] Yamamoto K . Five important preventive measures against exacerbation of coronavirus disease. Anaesthesiol Intensive Ther. 2021;53:358‐359. 35257568 10.5114/ait.2021.108581 PMC10165986

[16] Alberts B , Johnson A , Lewis J , Raff M , Roberts K , Walter P . Epidermis and its renewal by stem cells. Molecular Biology of the Cell. Fourth ed. Garland Science; 2002.

[17] Pastar I , Stojadinovic O , Yin NC , et al. Epithelialization in wound healing: a comprehensive review. Adv Wound Care. 2014;3:445‐464. 10.1089/wound.2013.0473 PMC4086220 25032064

[18] Yamamoto K . Adverse effects of COVID‐19 vaccines and measures to prevent them. Virol J. 2022;19:100. 35659687 10.1186/s12985-022-01831-0 PMC9167431

[19] Yamamoto K . Risk of heparinoid use in cosmetics and moisturizers in individuals vaccinated against severe acute respiratory syndrome coronavirus 2. Thromb J. 2021;19:67. 34530838 10.1186/s12959-021-00320-8 PMC8443895

[20] Gilbart MK , Jolles BM , Lee P , Bogoch ER . Surgery of the hand in severe systemic sclerosis. J Hand Surg Br. 2004;29:599‐603. 15542223 10.1016/j.jhsb.2004.03.013

